# Myliobatid Ray Gliding Dynamics: Experimental Tests of Body Shape and Tail Length on Stability

**DOI:** 10.1093/iob/obag002

**Published:** 2026-01-19

**Authors:** S B Cooper, C F White, G V Lauder, J Chaumel

**Affiliations:** Museum of Comparative Zoology, Harvard University, Cambridge, MA 02138, USA; Museum of Comparative Zoology, Harvard University, Cambridge, MA 02138, USA; Department of Environmental Sciences, University of Virginia, Charlottesville, VA 22903, USA; Museum of Comparative Zoology, Harvard University, Cambridge, MA 02138, USA; Museum of Comparative Zoology, Harvard University, Cambridge, MA 02138, USA

## Abstract

Myliobatid stingrays (eagle, cownose, and manta rays) swim using oscillatory locomotion, flapping their pectoral fins for propulsion while relying on their elongated tails for stability. During swimming, myliobatids often exhibit gliding behavior, a passive locomotion mode when active flapping ceases and the pectoral fins are maintained in a static position. We hypothesized that different pectoral fin conformations influence body stability and that the tail plays a critical role in stabilizing the models during gliding. To test this, we designed and 3D-printed four myliobatid-inspired models with different pectoral fin conformations: three with increasing dihedral angles and one model with an anhedral configuration. Each model was tested with three tail lengths: twice the disc width, equal to disc width, and no tail. Models were tested in a flow tank at increasing flow velocities. Stability, determined by pitch, roll, sway, and ODBA (overall dynamic body acceleration), was measured using high speed video and an accelerometer embedded into each model. When the models were compared without tails, the position of the pectoral fins also affected stability. Among models with dihedral angles, stability decreased with increasing dihedral angle. The model with an anhedral conformation was the most unstable. However, all models significantly reduced pitch, roll, sway, and ODBA with the presence of the tail, indicating that the tail had a stabilizing effect in all models regardless of the pectoral fin conformation. These findings indicate that pectoral fin conformation has a substantial effect on body stability and, in combination with the tail, enables stable passive gliding. Understanding the effect of body and pectoral fin posture on stability during locomotion is important for future efforts to analyze the energetic cost of locomotion and to understand the principles of efficient underwater movement.

## Resumen

Las rayas miliobatiformes (rayas águila, gavilanes y mantas) nadan mediante locomoción oscilatoria, batiendo sus aletas pectorales para generar propulsión y utilizando sus colas con forma de látigo alargadas para mantener la estabilidad. Durante la natación activa, los miliobatiformes alternan fases de batido con periodos de planeo, un modo de locomoción pasiva en el que cesa la oscilación y las aletas pectorales se mantienen orientadas hacia arriba en una posición estática. Nuestra hipótesis es que, durante el planeo, la conformación de las aletas pectorales influye en la estabilidad corporal de las rayas y que la longitud de la cola desempeña un papel fundamental en la estabilización bajo las distintas configuraciones de las aletas pectorales. Para testar esta hipótesis, diseñamos e imprimimos en 3D cuatro modelos de rayas inspirados en miliobatiformes en planeo, con diferentes conformaciones de las aletas pectorales: tres modelos con ángulos diedros crecientes y un modelo con una configuración anhedral. Cada modelo se ensayó con tres longitudes de cola: el doble del ancho del disco, igual al ancho del disco y sin cola. Los modelos se probaron en un canal de flujo a velocidades de corriente crecientes. La estabilidad del modelo, determinada a partir de las variaciones de cabeceo, balanceo, deriva lateral y ODBA (aceleración corporal dinámica total), se midió mediante un acelerómetro integrado en cada modelo y el análisis de vídeos de alta velocidad. Nuestros resultados muestran que, en ausencia de cola, la conformación de las aletas pectorales afecta a la estabilidad del modelo. Entre los modelos con ángulos diedros, la estabilidad disminuye a medida que aumenta el ángulo diedro, mientras que el modelo con configuración anhedral fue el más inestable. Sin embargo, todos los modelos redujeron significativamente el cabeceo, el balanceo, la deriva lateral y la ODBA en presencia de la cola, lo que demuestra su efecto estabilizador, independientemente de la conformación de las aletas pectorales. En conjunto, estos resultados indican que la conformación de las aletas pectorales tiene un efecto sustancial sobre la estabilidad corporal de las rayas miliobatiformes y que, en combinación con la cola, permite un planeo pasivo estable, con implicaciones para la eficiencia energética. Comprender el efecto de la postura de las aletas pectorales sobre la estabilidad durante la locomoción es clave para futuros estudios orientados a analizar el coste energético de la natación, además de a entender los principios del movimiento subacuático eficiente.

## Introduction

Locomotion is a vital, yet energetically costly activity for many organisms (Alexander 2005; Irschick and Higham 2016; Biewener and Patek 2018; Zhang and Lauder 2025). As a result, organisms have evolved a variety of energy-saving locomotion strategies, including soaring, drafting, and gliding (Pennycuick et al. 1975; Fish and Rohr 1999; Stöcker and Weihs 2001; Weihs 2004; Liao 2007; Gleiss, Jorgensen et al. 2011; Gleiss, Norman et al. 2011). During gliding, an animal maintains a relatively static body posture that minimizes drag and lowers metabolic costs (Thomas and Taylor 2001; Pennycuick 2008; Socha et al. 2015). Forward momentum originates from prior active locomotion (e.g., wing flapping, tail beats), but because drag is never fully eliminated, speed is gradually lost, ultimately limiting glide duration (Socha et al. 2015; Khandelwal et al. 2023). In negatively buoyant animals, however, glides can also be sustained by potential energy. As the animal sinks, forward motion arises from the combined effects of lift and buoyancy counteracting drag, enabling gliding to persist for extended periods (often >5 min) and substantially increasing energy savings (Pennycuick 2008; Gleiss, Jorgensen et al. 2011).

Gliding is associated with both aerial organisms like birds and aquatic animals like fishes and marine mammals (Weihs 1974; Fish and Rohr 1999; Stöcker and Weihs 2001; Thomas and Taylor 2001; Sachs 2005; Gleiss, Jorgensen et al. 2011; Gleiss, Norman et al. 2011). In pelagic marine systems, most gliders (including sharks, whales, and dolphins) generate thrust with their caudal fins or flukes during active swimming, a mode of locomotion known as axial propulsion (Fish and Rohr 1999; Wilga and Lauder 2004; Shadwick 2005; Gleiss, Jorgensen, et al. 2011; Zhan et al. 2017; Wainwright and Lauder 2020). These animals have a streamlined, fusiform body that helps them move forward efficiently through water, as well as possessing additional appendages (i.e., fins) that function as stabilizers during both active swimming and gliding (Fish and Lauder 2006). Batoids (rays and skates), in contrast, are dorsoventrally flattened and rely on their enlarged pectoral fins for propulsion. Other fins present in bony fishes such as the dorsal, anal, and pelvic fins, are reduced in batoids, limiting the stabilizing capacity of these structures.

**Fig. 1:**
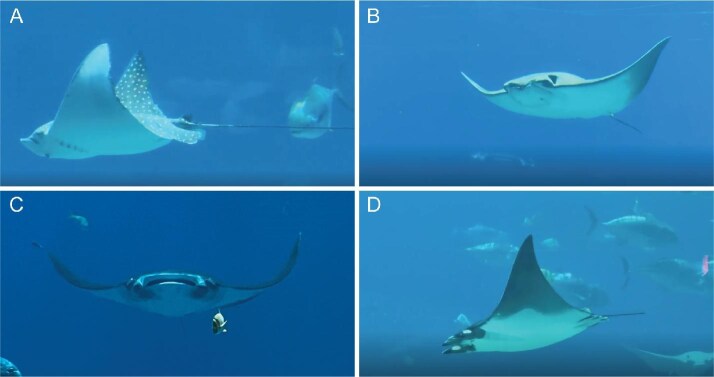
Myliobatid rays from different species gliding with the pectoral fins held at different dihedral angles. (A) Spotted eagle ray (*Aetobatus narinari*). (B) Cownose ray (*Rhinoptera bonasus*). (C) Giant manta ray (*Mobula birostris*). (D) Lesser devil ray (*M. hypostoma*). All images were taken by the authors at the Georgia Aquarium (USA).

Batoids exhibit substantial morphological diversity across species, which influences both lifestyle and swimming kinematics (Rosenberger 2001; Last 2016). The vast majority of batoids are benthic and rely on short wavelength (high frequency) undulations of their pectoral fins for thrust production. However, four families from the order of Myliobatiformes—Myliobatidae (eagle rays), Aetobatidae (pelagic eagle rays), Rhinopteridae (cownose rays), and Mobulidae (manta and devil rays) (Last 2016)—collectively referred to as myliobatids, produce thrust using long wavelength (low frequency) oscillations of their pectoral fins. The oscillatory locomotion in myliobatids is produced by highly specialized, large triangular pectoral fins that are stiffer and more laterally elongated than those of other batoids, while still retaining flexibility at the fin tips (Heine 1992; Fontanella et al. 2013; Hall et al. 2018). This morphology enables efficient oscillatory locomotion (active flapping) interspersed with short, intermittent glides (Rosenblum et al. 2011; Gao et al. 2023), as well as prolonged glides during vertical movements (Fontes et al. 2022). During gliding, myliobatids hold their pectoral fins in a relatively fixed dihedral position and curvature (Fig. 1; Supplementary Videos 1–3) (Rosenberger 2001; Parson et al. 2011; Fontanella et al. 2013; Braun et al. 2014; Fish et al. 2016; Fish et al. 2018; Gao et al. 2022, 2023). This pectoral fin posture has been proposed to enhance stability by limiting roll and yaw (Pennycuick et al. 1975; Weihs 1993; Webb and Weihs 1994; Fish 2004; Fish and Hoffman 2015; Wang et al. 2024) allowing the fins to function simultaneously as lift- and stability-generating surfaces. Despite these insights, the influence of pectoral fin posture and angle on gliding stability in rays has not yet been systematically tested.

Another key feature of myliobatid rays is the elongated whip-tail that extends posteriorly from the body (Last 2016; Chaumel and Lauder 2025). Myliobatid tails, which can be up to three times the body length (Bl) in some species, are relatively stiff, as they are supported by a fused vertebral column (Chaumel and Lauder 2025). Since myliobatids use their pectoral fins to generate thrust, the role of the tail in locomotion has been unclear. A previous study by Chaumel et al. (2025) demonstrated that adding a long slender tail can stabilize a towed myliobatid model. However, no studies have evaluated the interactive effect of tail length and pectoral fin posture on myliobatid swimming, particularly in the context of gliding behavior.

In this study, we focus on using four myliobatid body models with varying pectoral fin postures and tail lengths (1) to evaluate how different pectoral fin angles affect body stability during gliding, and (2) to investigate the interactive effect of tail length and fin posture on gliding stability by towing models in a flow tank over a range of speeds (Fig. 1). We tested three body models with the pectoral fins oriented at different dihedral angles (6.4°, 35.9°, and 80° concave upward), and one body model with the fins held at an anhedral angle (80° concave downward). Stability was assessed as the model’s ability to maintain a steady orientation and position under flow. Models displaying greater motions in pitch, roll, sway and overall dynamic body acceleration (ODBA) were interpreted as less stable, whereas models maintaining a steadier position (less motion) were considered more stable. We hypothesized that larger anhedral and dihedral fin angles are more unstable than lower angle fin surfaces. We also hypothesized that the presence of a tail would increase the stability of myliobatid models with unstable pectoral fin postures. This study will help elucidate how different positions of the pectoral fins affect gliding stability in myliobatid rays and how an elongate tail can act as a stabilizer. These results contribute valuable hydrodynamic and kinematic data to guide the future design of biomimetic gliding mechanical systems, as well as offer insights into the evolutionary and functional morphology of pelagic rays, contribute to understanding their swimming locomotion and behavioral adaptations to midwater environment and provide a comparative framework for studies of stability control across vertebrates.

## Materials and methods

### Design of myliobatid body models

**Fig. 2:**
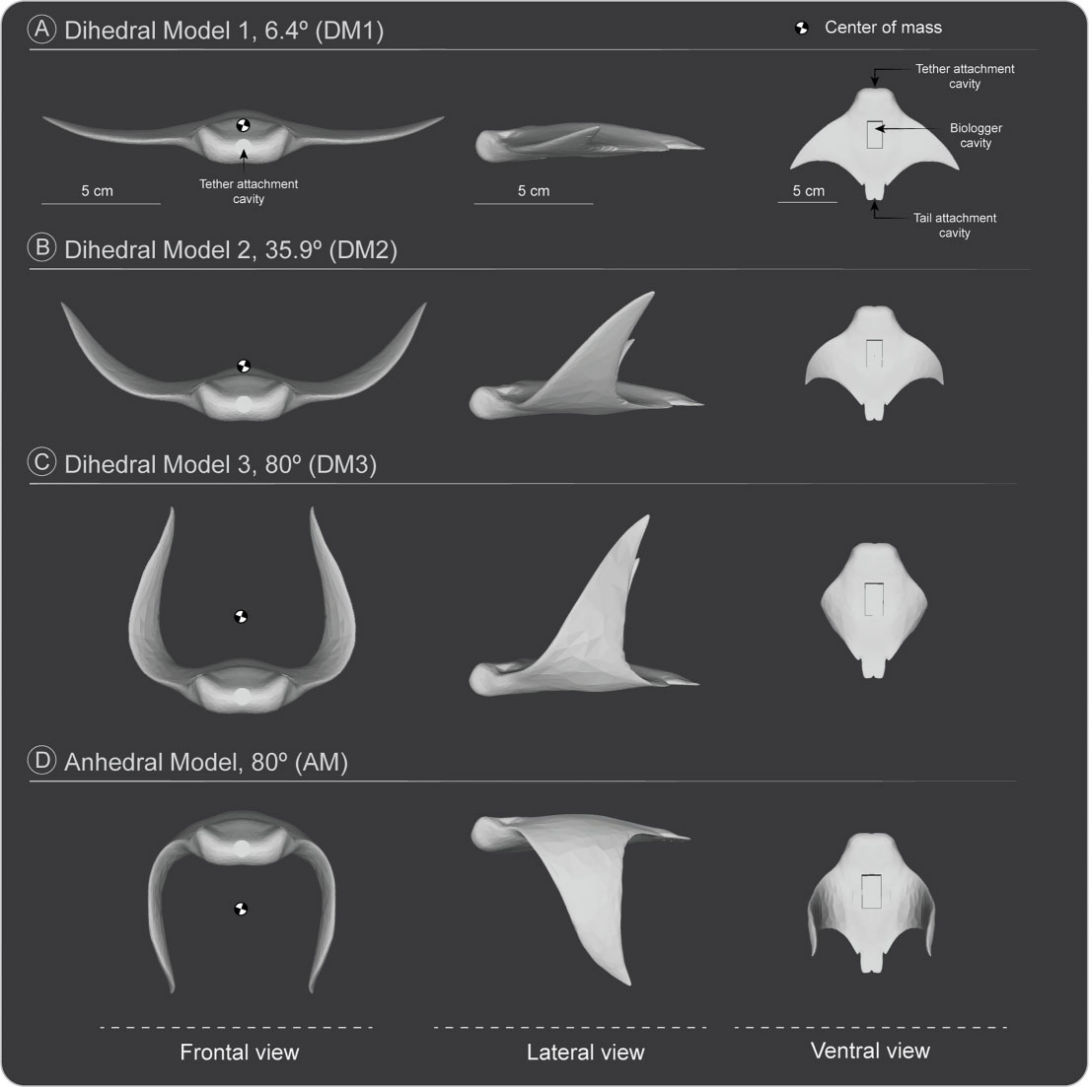
Anterior, lateral, and ventral views of the myliobatid ray models designed for hydrodynamic testing. (A) Model with a dihedral angle of 6.4° (DM1). Note the cavities made on all models to insert the biologger, the tether, and the tail. (B) Model with a dihedral angle of 35.9° (DM2). (C) Model with a dihedral angle of 80° (DM3). (D) Model AM with an anhedral angle of 80°. All models have disc widths of 15 cm and a Bl of 9.5 cm. The position of the COM (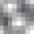) is approximated.

To test the effect of pectoral fin position on gliding performance, we designed four myliobatid ray models with varying pectoral fin postures using Autodesk Maya (version 2025.1; Autodesk Inc., USA) (Fig. 2). All models were derived from a baseline configuration developed and tested in a previous study (Fig. 2A) (Chaumel et al. 2025). Briefly, this baseline model (with pectoral fins extended) was designed to represent a “generalized” myliobatid morphology based on three-dimensional models available online (Turbosquid 2023; DaRocha 2024). Models were altered to more accurately reflect the morphology of myliobatids, including the modification of several anatomical features (e.g., body thickness, shapes of the pectoral and pelvic fins). Models were also altered to be symmetrical by creating one half and mirroring it to form the other half. Thus, all models followed similar body morphometrics, including 15 cm disc width (distance between pectoral fin tips), Bl of 9.5 cm, and equivalent surface area and volumes (14,201 total surface area mm^2^; 42,242 volume mm^3^). Models were passive, without any actuation mechanisms.

Three models were designed with the pectoral fins at a dihedral angle (i.e., in a concave upward configuration) to mimic pectoral fin configurations of rays during gliding, while one model was designed with the pectoral fins at an anhedral angle (i.e., in a concave downward configuration) (Fig. 2; Supplementary Models 1–4). Dihedral models are abbreviated as DM and the anhedral model is abbreviated as AM. To determine a realistic fin gliding position, the pectoral fin elevation angle was measured in a total of 10 gliding myliobatids of different species using online images (Supplementary Table 1). Angles were measured with Image J (National Institute of Health, USA) using both sides of the animal. The right and left values were then averaged, and the mean was taken to yield the final fin angle measurement. The average fin elevation angle of surveyed gliding rays was 35.5°.

Model DM1 (dihedral model 1) was designed with the pectoral fins laterally extended, with slight extension above the vertical plane at the fin tips (Fig. 2A, Supplementary Model D1), at a gliding angle of 6.4°. Model DM2 (dihedral model 2) was designed to resemble the body posture of myliobatids during gliding (Figs. 1 and 2B) with a fin angle of 35.9° (Supplementary Model D2). Model DM3 represents an extreme dihedral fin posture (Fig. 2C) where the fin elevation angle of DM3 was set at 80° (Supplementary Model D3). Finally, model AM was constructed with an extreme pectoral fin anhedral angle of 80° (Fig. 2D, Supplementary Model AM). The AM model was included in this study to assess the effect of anhedral fin postures on gliding stability, although we have not observed a myliobatid species gliding with this posture/fin position.

Three cavities were created in all models: one centered in the anterior surface of the model to attach the tether, one at the center of the ventral surface to insert an acceleration datalogger (details below), and one centered in the posterior end between the pelvic fins to insert the tail (Fig. 2). Each model was printed with a FormLabs Form 3 + printer using Elastic50A V2 photopolymer resin (FormLabs Inc., USA). This material was chosen because its density (1.1 g/mL^3^) is comparable to that of the density measured for myliobatids (Heine 1992; Fish and Hoffman 2015; Chaumel et al. 2025).

Models were tested using tails with three different lengths: (1) 30 cm tail, equal to double the model disc width (2:1), (2) 15 cm tail, equivalent to the model disc width (1:1), and (3) no tail (0:1). Tails were made from a NinjaFlex thermoplastic polyurethane filament of 0.3cm in diameter (NinjaTek, USA) due to its similar shape and density to myliobatid tails. The full-length tail represented 2.5% of body mass. Before each trial, a 30 cm tail was inserted into a cavity located at the posterior end of the body, between the pelvic fins. We marked the tail at 15 cm with a blue line to guide cutting at this location during testing.

### Flow tank testing

Stability is typically divided into two components: static and dynamic stability. Static stability describes the initial response of an object immediately after a disturbance (such as change in flow velocity). An object with positive static stability resists the disturbance and tends to return toward its original orientation, whereas an object with negative static stability will fail to return to equilibrium. Dynamic stability describes the time-dependent behavior of the object following a disturbance, characterizing whether oscillations grow, stay the same, or decay. An object with positive dynamic stability damps these oscillations and returns to equilibrium, while an object with negative dynamic stability exhibits increasing oscillations that move it further from its original orientation. If oscillations remain constant with time, the object is considered to have neutral dynamic stability.

In classical stability analyses, an untethered object is perturbed, and the resulting forces and motions are used to infer static and dynamic stability. In our experiments, variations in flow speed and minor deviations from laminar flow were interpreted as continuous perturbations. However, we did not measure the models’ immediate reaction to a given perturbation (static stability), but rather how they moved over time, comparing the extent of motion among models with different tail lengths. Because we did not quantify how oscillations increased or decayed over time for a specific disturbance, we are not directly measuring dynamic stability. Instead, we defined stability as the degree of motion exhibited by each model. Models showing greater roll, pitch, sway, and ODBA were interpreted as less stable, whereas those showing smaller movements were considered more stable. The tethering of the models also imposes limitations on our interpretation of stability, as it constrains translational movement and inherently provides a restoring force that conditions the model to return toward its original position.

**Fig. 3:**
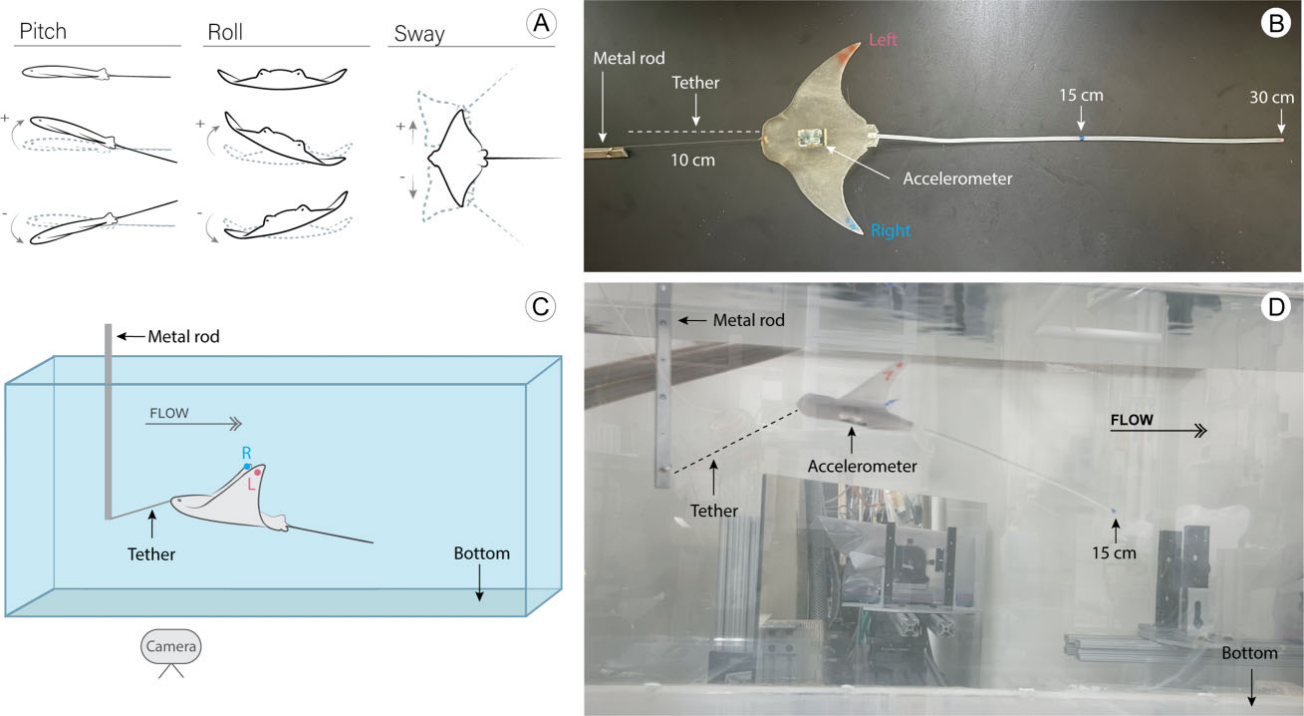
Variables measured and experimental setup. (A) Motion variables measured in the model: pitch (up–down rotation), roll (lateral rolling motion), and sway (side-to-side displacement). (B) Model DM2 attached to the metal rod via a tether (fishing line), with a 30 cm tail. Tail lengths of 30 cm and 15 cm are indicated by marks along the tail (red for 30 cm, blue for 15 cm). The accelerometer is positioned at the COM. (C) Experimental setup in the flow tank. (D) Model DM2 mounted in the flow tank during the experiment.

To assess how fin posture and tail lengths affect model stability, models were tested in a recirculating tank at increasing flow speeds (Fig. 3). In the flow tank, all models were towed using a 10 cm, 3.6 kg (eight-pound) monofilament line attached to the rostrum from a rigid support (Fig. 3). This ensured that the model held streamwise position in the flume over the range of tested speeds and allowed both video and data logger measurements to assess movement of the models.

There are a variety of possible protocols for testing models and robotic systems, some of which use a rigid support attached to models (Tangorra et al. 2008; Tangorra et al. 2011; Esposito et al. 2012), while others use an attached line to tow models or fish to quantify drag (Drucker and Lauder 1999). Using a towed line corresponded to the testing protocol and dynamics analysis used for understanding kite aerodynamics and stability (Marvin 1897; Borobia‐Moreno et al. 2021) as well as standard ship model tow testing protocols (Papoulias 1992; Fitriadhy et al. 2013). Stability analyses can be accomplished using a theoretical framework or experimental means (e.g., models rigidly attached as in ship or airplane testing). Our protocol represents a compromise between allowing free motion of the body and limiting motion with a rigid support which reduces the information that can be collected by a data logger placed on the model.

These tests were designed to provide a comparative examination of the stability of alternative body models at a size amenable to experimental evaluation and not to mimic natural myliobatid ray movements or actual size. The towed testing protocol does impact various possible body movements and will necessarily limit changes in pitch within each speed due to the attachment of the towing line at the front of the model. However, changes in pitch across models and speeds can still be analyzed and the towed protocol does not impact roll stability as the models are free to rotate in that dimension (Chaumel et al. 2025).

**Fig. 4:**
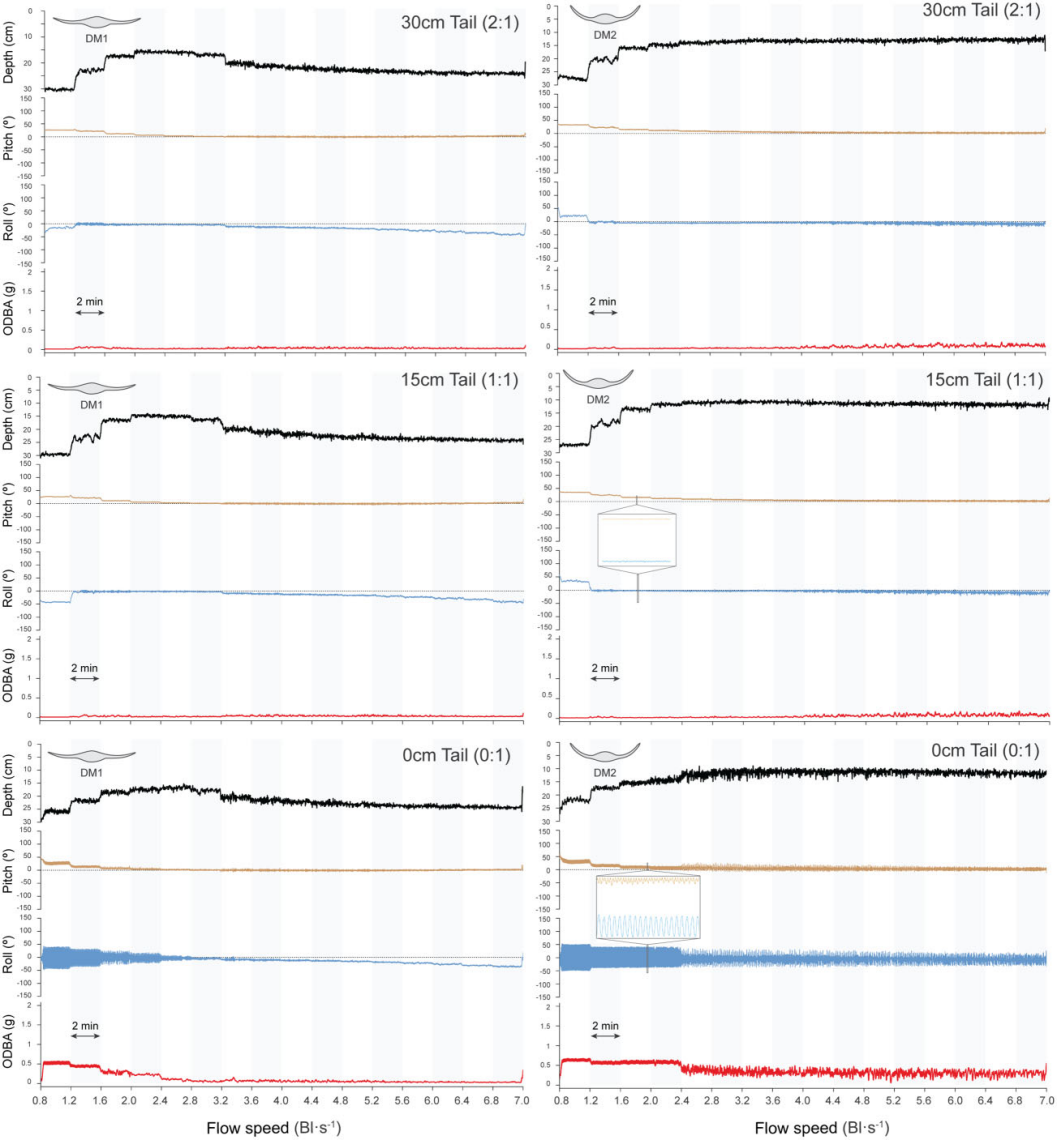
Depth (in the flow tank), body pitch, body roll, and ODBA at each tail length for DM1 and DM2. Results for DM1 are shown on the left: 30 cm tail (2:1), top; 15 cm tail (1:1), middle; and 0 cm tail (0:1), bottom. Results for DM2 are shown on the right in the same order. Variations in each measured variable (depth, pitch, roll, and ODBA) are shown as flow speed increases every 2 min (gray and white columns). Positive pitch values indicate head-up orientation, while negative values indicate head-down orientation. Positive roll values correspond to the right pectoral fin tip elevated above the left, whereas negative values indicate the opposite. Graphs shown for each model correspond to the results observed in 1 trial (the experiment consisted of a total of 3 trials). Note the zoomed areas of the oscillation patterns for models without tail.

Each model was tested, at each tail length, a total of 3 times (trials). A trial began with a model with a 30 cm tail (Fig. 4B). Once the model was settled and motionless in the tank, the flow was turned on at an initial speed of 0.8 Bl s^−1^ (body lengths per second) or 7.5 cm/s. The flow rate was then gradually increased by 0.4 Bl s^−1^ every two minutes, finishing at 6.0 Bl s^−1^ or 56.7 cm/s. Upon reaching 6.0 Bl s^−1^, the flow was then turned down to 0 Bl s^−1^, and the tail was cut to 15 cm in length (at the previously added mark). The same process was repeated for the same model with a 15 cm tail length. After completing these recordings, the tail was reduced to 0 cm, and the entire experiment was repeated. All trials were recorded from a lateral view using an iPhone 13 to provide a visual record of the experiment (Supplementary Videos 4–7). These videos were used posteriorly as a visual reference of the accelerometer data, helping to identify unexpected events (e.g., tether breaks, model spins), as well as to align starting and ending times. The Reynolds number of the experimental tests ranged from approximately 7000 to 23,000 (using model Bl). This is substantially lower than Reynolds numbers for large gliding myliobatid rays, which can be in the millions.

### High-speed camera recording

Separate from the accelerometer experiments, additional trials were conducted to measure sway. The ventral view of each model was recorded in the flow tank at various speeds using a Photron FASTCAM Mini AX50 high-speed camera (Photron Inc., USA) operating at 125 fps. Recordings were obtained for all models with each tail length (30 cm, 15 cm, and no tail) across flow speeds ranging from 1.5 BL s⁻¹ (14.1 cm s⁻¹) to 6.0 BL s⁻¹ (56.7 cm s⁻¹) in increments of 0.75 BL s⁻¹ (3.3 cm s⁻¹). Footage was processed in Photron FASTCAM Viewer 4 (Photron Inc., USA) to quantify sway. Each video was analyzed frame by frame, and sway amplitude (i.e., lateral displacement of the model) was measured as the maximum lateral displacement of the model at its anterior surface. Sway amplitude was calculated three times for each model and each flow speed, and the mean was used as the final sway amplitude value for each speed and model.

### Data analysis and statistics

Three-dimensional acceleration data obtained from the embedded datalogger (Axy-5, TechnoSmart inc., Italy) were used to calculate pitch, roll, and ODBA. Data visualization and statistical analysis were completed using Igor Pro (Wavemetrics Inc., USA, Version 8.04) and R (Version 4.4.2, R Core Team). These dataloggers are factory calibrated and recorded proper acceleration, which is acceleration relative to free-fall, at 100 Hz, with 10-bit resolution and a ±2G range. Proper acceleration is a combination of the acceleration due to the rotational and translational movements of the sensor, as well as acceleration due to gravity (static acceleration), which can be used to indicate orientation (pitch and roll) (Whitney et al. 2018). The model’s pitch (rotation about the medial-lateral axis) was estimated using EQ2, and the model’s roll (rotation about the anterior-posterior axis) was estimated using EQ3. Raw acceleration data were used when calculating orientation to create the most temporally responsive estimates, and preliminary analysis showed little variation when the acceleration data was low-pass filtered.



\begin{eqnarray*}
{\rm EQ2.}\ {\mathrm{Pitch}} = {\mathrm{atan}}( {\textit{accX},\textit{AccZ}} ),\\
{\rm EQ3.}\ {\mathrm{Roll}} = {\mathrm{atan}}2( {\textit{accY},\textit{accZ}} ),\\
{\rm EQ4.}\ {\mathrm{Mean}}\,{\mathrm{Roll}} = {\mathrm{atan}}2( \textit{mean}(\cos ( {\textit{roll}} ),\\
\quad \textit{mean}(\sin (\textit{roll}) ),\\
{\rm EQ5.}\ {\mathrm{ODBA}} = {\mathrm{abs}}( {acc{X}_{dyn}} ) + {\mathrm{abs}}( {acc{Y}_{dyn}} )\\
\quad + {\mathrm{abs}}( {acc{Z}_{dyn}} ).\end{eqnarray*}

In addition to acceleration due to gravity, proper acceleration also contains dynamic acceleration, or the acceleration due to the translational movements and postural changes of the model. Dynamic acceleration was isolated as the deviations in the raw acceleration data from a 3 s running mean of the raw acceleration data for each axis (Shepard et al. 2008). ODBA was calculated as the sum of the absolute value of the *X*, *Y*, and *Z* axis dynamic acceleration at each time point (Wilson et al. 2006; Gleiss, Wilson et al. 2011). ODBA is a comprehensive, continuous metric that integrates roll, pitch, and yaw without requiring video analysis. Commonly used in biologging to study animal movement and estimate metabolic rate (Wilson et al. 2006; Gleiss, Jorgensen et al. 2011), using ODBA here enabled rapid quantification of how body shape and tail length affect stability (i.e., model movement) during tow tests, providing a reliable estimate of model stability.

Each flow condition during a trial was isolated and the following summary statistics were calculated: pitch and roll mean, pitch and roll range, and mean ODBA. Pitch range was calculated as the difference between the maximum and minimum pitch experienced during a flow condition and thus can be a maximum of 180°. As roll is a circular metric (−180° through 180°), we first had to calculate the circular mean roll position of the model (EQ4). Roll range was calculated as the difference between the maximum and minimum roll experienced in reference to the model’s mean position during a flow condition and could be a maximum of 360°. Since no myliobatid models were able to elevate from the bottom at 0.8 Bl s^−1^ this experimental flow speed has been excluded from data analyses and statistics.

In order to compare how tail length, pectoral fin angle (model type), and flow speed impacts stability metrics (pitch range, roll range, ODBA, and sway), a series of linear models were constructed. Throughout all statistical models, tail length, pectoral fin angle (model type), and flow speed were considered categorical factors. To increase interpretability of results models were limited to two predictor variables at a time. To understand the influence of tail length and flow speed, separate models were run for each pectoral fin configuration (myliobatid model) (see Supplementary Table 2). Subsequently to understand the effect of pectoral fin angle, separate models were constructed for each tail length, with flow speed and myliobatid model type (pectoral fin angle) as the predictor variables (see Supplementary Table 9). All models contained the interaction between the two predictor variables, and significance was determined using an analysis of variance (ANOVA). After initial model construction, post hoc pairwise comparisons were conducted, using a *T*-test. Post hoc comparisons were planned beforehand and only compared which tail length and pectoral fin angle models differed within each speed (see Supplementary Tables 3–6 and 10–15). Statistical significance was set at *P* < 0.05 and was adjusted using a Bonferroni correction for the post hoc tests. Throughout the manuscript, results are presented as mean ± standard deviation, unless otherwise noted, and variance in figures is presented using ± 2*standard error.

## Results

**Fig. 5:**
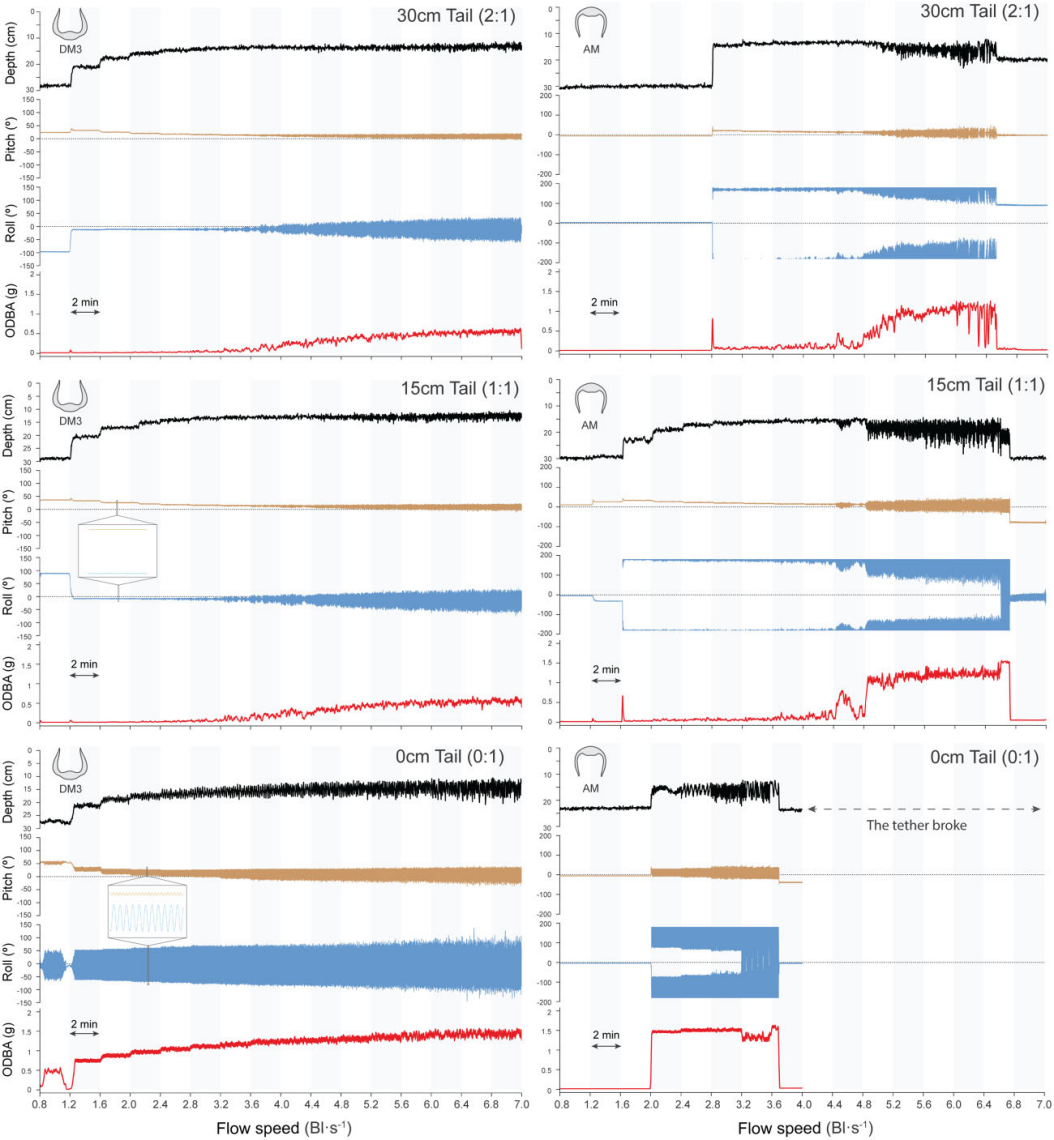
Depth (in the flow tank), body pitch, body roll, and ODBA at each tail length for DM3 and AM. Results for DM3 are shown on the left: 30 cm tail (2:1), top; 15 cm tail (1:1), middle; and 0 cm tail (0:1), bottom. Results for AM are shown on the right in the same order. During experiments with AM, the tether broke due to extreme model instability and the trial was stopped; after 2 BL s⁻¹, the model performed rotational flips around the tether point. Variations in each variable (depth, pitch, roll, and ODBA) are shown as flow speed increased every 2 min (gray and white columns). Positive pitch values indicate head-up orientation, while negative values indicate head-down orientation. Positive roll values correspond to the right pectoral fin tip elevated above the left, whereas negative values indicate the opposite. Graphs shown correspond to a single representative trial (three trials were conducted per model). Note the zoomed areas of the oscillation patterns for models without tail.

**Fig. 6:**
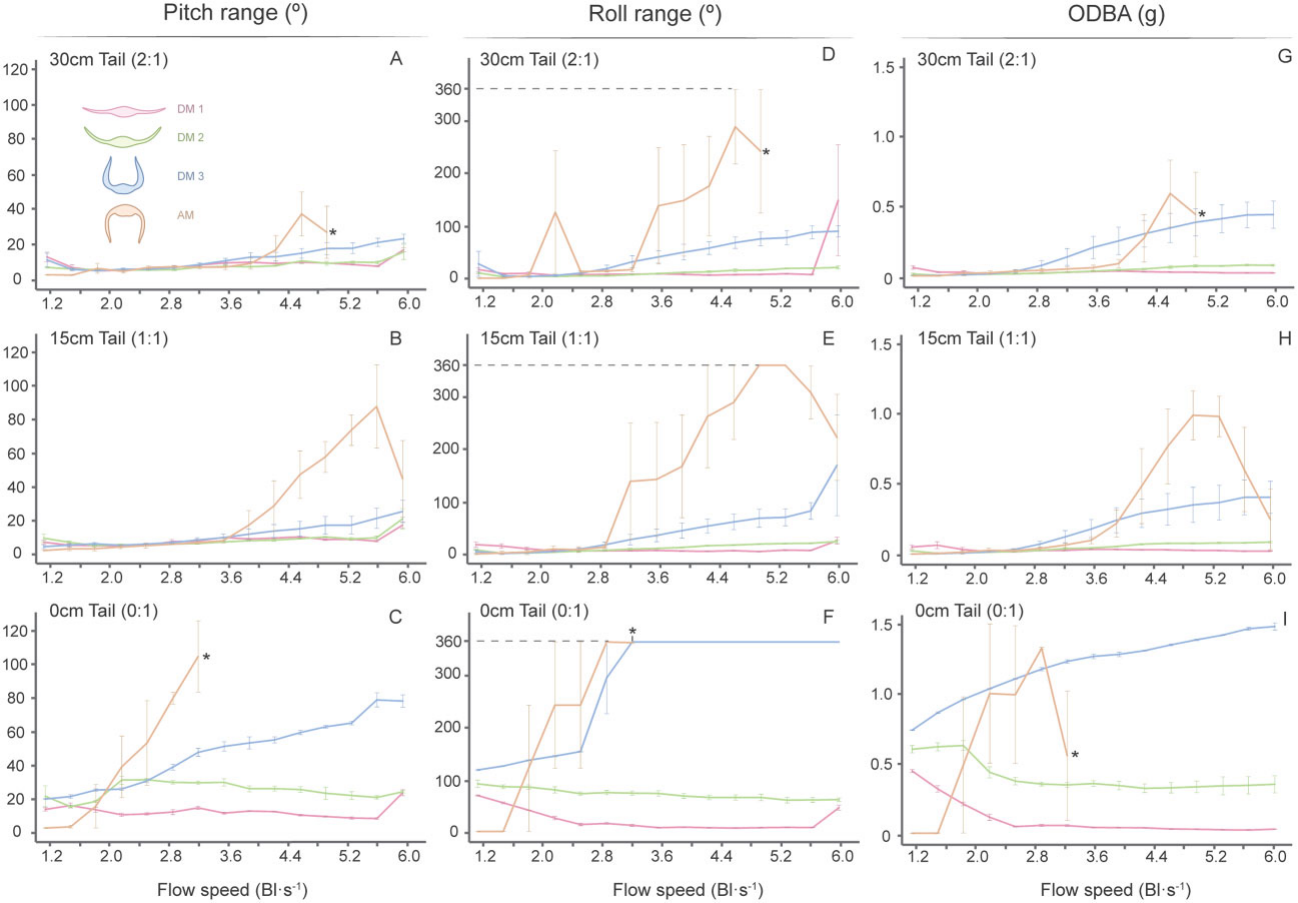
Mean pitch range, roll range, and ODBA values for each model and tail length plotted against flow speed. Mean pitch range represents the maximum pitching displacement recorded at a given flow speed, averaged across trials. Mean roll range represents the maximum rolling displacement under the same conditions. Mean ODBA is the sum of the absolute dynamic accelerations in the *x*, *y*, and *z* axes, averaged across trials. (A–C) Mean pitch range for tail lengths of 30, 15, and 0 cm, respectively. (D–F) Mean roll range for tail lengths of 30, 15, and 0 cm. (G–I) Mean ODBA for tail lengths of 30, 15, and 0 cm. Asterisk (*) associated with AM indicates the flow speed at which the tether broke due to extreme model instability, and the experiments were stopped. Dashed line in roll at 360° indicates the maximum value possible for roll (full loop). Colored inset icons illustrate the four tested pectoral fin postures that correspond to the graph colors.

Pitch range, roll range, sway, and ODBA were measured to evaluate the movement and stability of the tethered myliobatid ray models across varying pectoral fin angles, flow speeds, and tail lengths (Figs. 4–6).

### The effect of pectoral fin posture on stability

**Fig. 7:**
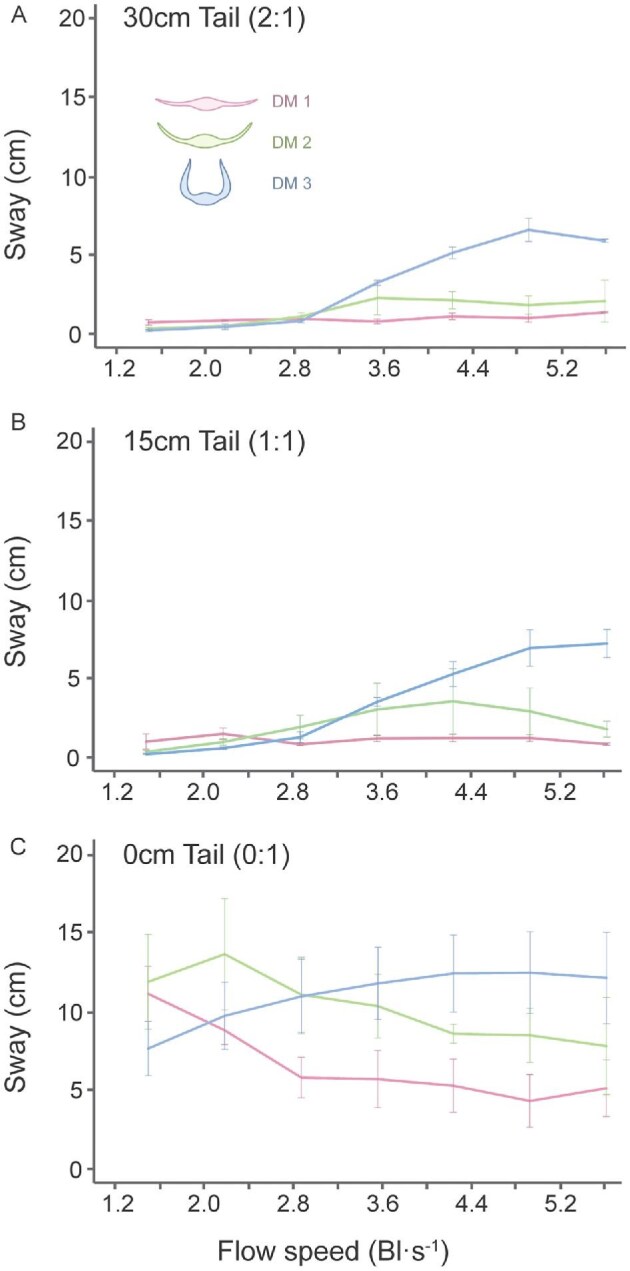
Sway range across models and tail lengths at increasing flow speeds. Mean sway range represents the average maximum lateral displacement of the model (left–right) in the tank for a given flow speed. (A) Models with 30 cm tail length. (B) Models with 15 cm tail length. (C) Models with 0 cm tail length. Colored inset icons indicate the different models tested, each with different pectoral fin postures, matching the graph colors. AM models were excluded from this analysis.

**Fig. 8:**
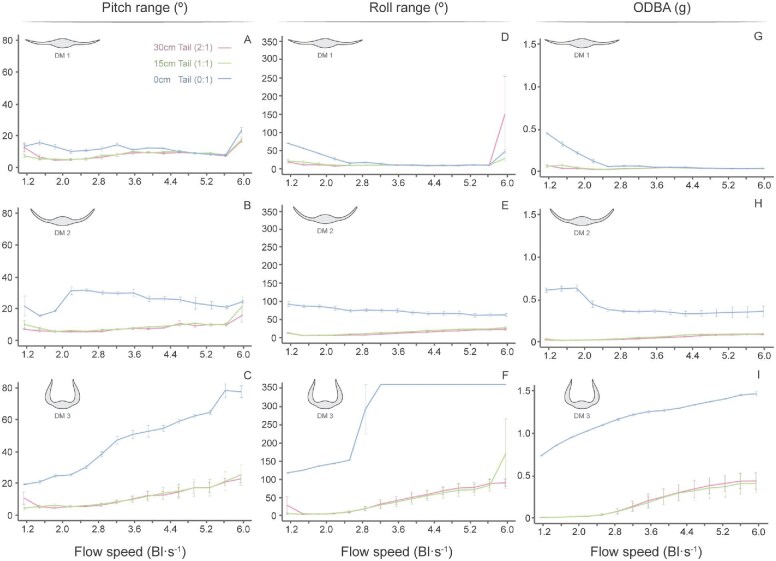
Mean pitch range, roll range, and ODBA values for each model and tail length plotted against flow speed. Mean pitch range represents the maximum pitching displacement recorded at a given flow speed, averaged across trials. Mean roll range represents the maximum rolling displacement under the same conditions. Mean ODBA is the sum of the absolute dynamic accelerations in the *x*, *y*, and *z* axes, averaged across trials. Data for AM was excluded due to instability that caused the model to spin, ultimately causing the tether to break. (A–C) Mean pitch range for DM1, DM2, and DM3 at each tail length. (D–F) Mean roll range for DM1, DM2, and DM3. (G–I) Mean ODBA for DM1, DM2, and DM3. Inset icons illustrate the tested pectoral fin posture.

Pectoral fin posture had a significant influence on pitch range, roll range, ODBA, and sway. Tailed dihedral models (DM1, DM2, and DM3) did not differ significantly in pitch range, roll range, ODBA, and sway across most speeds (*t*-test, *P* > 0.05; Figs. 7, 8; Supplementary Tables 2–6). However, in the absence of a tail, dihedral models yielded significantly different pitch, roll, and ODBA values across most flow speeds (*t*-test, *P* < 0.05; Fig. 8; Supplementary Tables 2, 7, and 8).

Among the tailless dihedral models, DM3 exhibited the greatest pitch range, roll range, and ODBA values (Pitch Range: DM3_0:1_ x̄ = 47.5° ± 19.8; Roll Range: DM3_0:1_ x̄ = 280.8° ± 107.6; ODBA: DM3_0:1_ x̄ = 1.21g ± 0.22), followed by DM2 (Pitch Range: DM2_0:1_ x̄ = 24.9° ± 4.8; Roll Range: DM2_0:1_ x̄ = 73.5° ± 9.7; ODBA: DM2_0:1_ x̄ = 0.44g ± 0.11) and DM1 (Pitch Range: DM1_0:1_ x̄ = 12.5° ± 3.8; Roll Range: DM1_0:1_ x̄ = 24.2° ± 20.3; ODBA: DM1_0:1_ x̄ = 0.11g ± 0.13) (Figs. 6 and 8; Table 1). This suggests that larger dihedral fin angles are inherently less stable. Sway was the only stability metric that did not adhere to this trend, while models with larger dihedral angles maintained a lower overall sway amplitude. These differences were not significant at any individual speed (*t*-test, *P* > 0.05; Fig. 7; Supplementary Tables 2 and 8).

**Table 1:** Mean values for pitch range, roll range, and ODBA across speeds and tail lengths for the myliobatid models

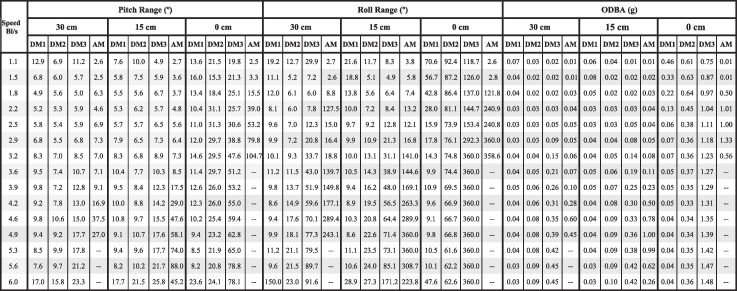

The AM, with downward-angled pectoral fins, exhibited the greatest instability among all models. As soon as the model was able to generate sufficient lift to take off from the bottom of the tank, the model immediately rolled 180° and thus established a “fin up” posture similar to DM3 (Supplementary Video 7). AM did not yield significantly higher pitch, roll, and ODBA values than DMs at most speeds (*t*-test, *P* > 0.05; Fig. 6; Supplementary Tables 2–8). However, the specific flow speeds at which AM lifted varied between trials, resulting in large SE values (Fig. 6), and limiting the statistical power for comparisons with DMs. In addition to immediately rolling 180°, as the flow speed increased, the roll range of AM increased. In two of the three trials, the model displayed repeated rolls (>360°) to each side and began to spin around the tether attachment. This spinning motion increased tension on the towline, resulting in rupture at approximately 3 Bl s^−1^ (although the precise breaking speed varied across trials) (asterisk in Fig. 6C, F, and I, Supplementary Video 7). Measuring sway was not possible for this model, as the continuous flipping rendered the lateral displacement of AM negligible.

### The effect of the tail on stability

The presence of a tail significantly reduced the motion of all myliobatid models tested. Models with a tail consistently displayed lower pitch ranges, roll ranges, ODBA, and swaying amplitude (lateral motion) (Figs. 6–8; Supplementary Tables 9–15). However, the length of the tail (1:1 vs. 2:1) had no significant effect on any of these metrics in any of the models, indicating that both tail lengths provided equivalent stabilization (*t*-test, *P* > 0.05; Figs. 6–8; Supplementary Tables 9–15). Flow speed also had a significant impact on model’s motion, with models displaying an elevated ODBA and increased pitching and rolling motions at faster speeds (Figs. 6–8; Supplementary Table 2 and 9). However, the influence of the tail and flow speed was not uniform across models (Figs. 6–8).

Among all models, DM1 showed the most comparable behavior between its tailless and tailed configurations (Figs. 6–8). DM1 displayed minimal differences in pitch range across all tail lengths and flow speeds (<6 Bl s^−1^), with values ranging from 4.9 to 16.0° (Fig. 8A; Table 1). In contrast, roll range and ODBA values were reduced by the presence of the tail.

In general, DM1 exhibited significantly higher roll and ODBA values in the absence of a tail (Roll Range: ANOVA *F*_2,141_ = 16.4, *P* < 0.05; ODBA: *F*_2,141_ = 18.4, *P* < 0.05; *t*-test, *P* < 0.05; Supplementary Tables 14–15). However, there was a significant interaction between tail length and flow speed (Roll Range: *F*_30,96_ = 2.1, *P* < 0.05; ODBA: *F*_30,96_ = 136.3, *P* < 0.05; Supplementary Table 9). With a tail, DM1 maintained low roll and ODBA values across all speeds (Roll Range: DM1_2:1_ x̄ = 10.7° ± 2.7, DM1_1:1_ x̄ = 11.7° ± 3.9; ODBA: DM1_2:1_ x̄ = 0.04g ± 0.01, DM1_1:1_ x̄ = 0.04g ± 0.01; Fig. 8D and G; Table 1). Tailless DM1 displayed a marked decrease in roll and ODBA between 1.1 Bl s^−1–1^ and 2.5 Bl s^−1^, subsequently yielding values comparable to tailed configurations (Roll Range at and above 2.5 Bl s^−1^: DM1_0:1_ x̄ = 15.0° ± 3.0; ODBA at and above 2.5 Bl s^−1^: DM1_0:1_ x̄ = 0.05g ± 0.01; *t*-test, *P* > 0.05; Fig. 8D and G; Table 1; Supplementary Tables 9–10). As such, tailless DM1 only yielded significantly higher values than tailed configurations at flow speeds below 2.5 Bl s^−1^ (Roll Range below 2.5 Bl s^−1^: DM1_0:1_ x̄ = 49.5° ± 18.3; ODBA below 2.5 Bl s^−1^: DM1_0:1_ x̄ = 0.29g ± 0.14; *t*-test, *P* < 0.05; Fig. 8D and G; Table 1; Supplementary Tables 9–10). The sway data for this model showed a similar pattern; sway values for tailless DM1 did not decline to the level of tailed configurations; tailless DM1 sway was approximately five times higher than DM1 with a tail at speeds above 2.5 Bl s^−1^ (Fig. 7).

The stabilizing effect of the tail was more pronounced in DM2 than in DM1, particularly with regards to pitch range, roll range, and ODBA. Overall, tails significantly reduced pitching movements (ANOVA *F*_2,141_ = 196.5, *P* < 0.05; *t*-test, *P* < 0.05; Supplementary Tables 14–15), with tailed DM2 producing pitch range values three times lower than the tailless configuration (Fig. 8B). However, speed has a significant effect. DM2 displayed increased pitching motions without a tail; and pitch range increased from 1.1 to 2.5 Bl s^−1^ (Fig. 8B). Beyond this speed, pitch range declined, ultimately reaching values just over 20° (Fig. 8B).

Roll range and ODBA were also significantly reduced by the presence of a tail for DM2 (ANOVA, Roll Range: *F*_2,141_ = 589.2, *P* < 0.05; ODBA: *F*_2,141_ = 348.6, *P* < 0.05; *t*-test, *P* < 0.05; Fig. 8E and H; Supplementary Tables 14–15). As with DM1, DM2 did not exhibit a significant difference in roll range and ODBA between the 15 and 30 cm tail lengths (Roll Range: DM2_2:1_ x̄ = 13.0° ± 6.2, DM2_1:1_ x̄ = 15.4° ± 7.2; ODBA: DM2_2:1_ x̄ = 0.05g ± 0.03, DM2_1:1_ x̄ = 0.06g ± 0.03) (*t*-test, *P* > 0.05; Fig. 8E and H; Table 1; Supplementary Tables 14–15). Without a tail, DM2 roll range and ODBA values decreased as flow speed increased (Fig. 8E). Roll range declined gradually from 92.4° at 1.1 Bl s^−^^1^ to 62.6° at 6 Bl s^−^^1^ (Fig. 8E; Table 1). ODBA dropped sharply from 0.64 g at 1.8 Bl s^−^^1^ to 0.38 g at 2.5 Bl s^−^^1^, subsequently plateauing at faster speeds (Fig. 8H; Table 1). On average, tailless DM2 yielded roll range and ODBA values 5.2 and 7.5 times higher than tailed configurations, respectively (Roll Range: DM2_0:1_ x̄ = 73.5° ± 9.7; ODBA: DM2_0:1_ x̄ = 0.41g ± 0.11; Fig. 8E and H; Table 1). Roll range and ODBA were significantly higher for tailless DM2 than for tailed configurations at flow speeds below 5.3 Bl s^−^^1^ (*t*-test, *P* < 0.05; Supplementary Tables 9 and 11). As with tailless DM1, sway in tailless DM2 was higher than the tailed configurations and decreased as a function of flow speed. However, unlike DM1, sway in DM2 with tails increased with flow speed (Fig. 7).

Among the DMs, the presence of a tail improved stability most in DM3, as evinced by the marked differences in pitch, roll, ODBA, and yaw values between tailless and tailed configurations of DM3 (Figs. 7 and 8C, F, and I). Tailless DM3 was more unstable than tailed configurations in all stability metrics and for all flow speeds (Figs. 7 and 8C, F, and I). DM3 pitched 3.9 times more, on average, in the absence of a tail (Pitch Range: DM3_2:1_ x̄ = 12.0° ± 5.9, DM3_1:1_ x̄ = 12.1° ± 6.5, DM3_0:1_ x̄ = 47.5° ± 19.8; Table 1) (ANOVA, *F*_2,141_ = 103.9, *P* < 0.05; *t*-test, *P* < 0.05; Supplementary Tables 14–15) and pitch range increased with flow speed for all tail lengths (Fig. 8C).

Roll range was the stability metric most affected by the presence of the tail for DM3. Tailed DM3 roll range increased with flow speed from 4.9 to 171.2° (Fig. 8F; Table 1). Roll range increased gradually for tailless DM3 between 1.1 and 2.5 Bl s^−^^1^, but between 2.5 and 3.2 Bl s^−^^1^ roll rose dramatically, reaching 360° (the maximum value) indicating the model would fully flip upside down at least temporarily. (Fig. 8F; Table 1). This extreme roll range value persisted at all subsequent flow speeds (Fig. 8F). As such, DM3 produced roll range values over 200° greater, on average, without a tail (Roll Range: DM3_2:1_ x̄ = 45.4° ± 31.2, DM3_1:1_ x̄ = 46.8° ± 44.0, DM3_0:1_ x̄ = 280.8° ± 107.6; Fig. 8F; Table 1) (ANOVA, *F*_2,141_ = 182.2, *P* < 0.05; Supplementary Tables 14–15).

The stabilizing effect of the tail on DM3 was also observed in ODBA; tailless DM3 yielded ODBA values 5.9 time higher, on average, than tailed configurations (ODBA: DM3_2:1_ x̄ = 0.21g ± 0.17, DM3_1:1_ x̄ = 0.20g ± 0.16, DM3_0:1_ x̄ = 1.21g ± 0.22) (ANOVA *F*_2,141_ = 248.3, *P* < 0.05; *t*-test, *P* < 0.05; Supplementary Tables 14–15). ODBA gradually increased with flow speed for all tail lengths (Fig. 8I). In contrast, sway increased much more as a function of flow speed for DM3 with a tail than it did for tailless DM3, which maintained an elevated sway (Fig. 7).

Of all models, AM recorded the most dramatic differences in movement between tailless and tailed configurations. Without a tail, AM pitched and rolled to such an extreme degree that it began to repeatedly flip, eventually rupturing the towline (asterisk in Fig. 6C, F, and I). Despite being inherently unstable and flipping upon lifting, no AM configuration with a tail exhibited this behavior. Similarly to DM3, AM yielded higher pitch range, roll range, and ODBA values as flow speed increased, but, due its extreme, violent movements, the precise variation of these stability metrics did not vary significantly between flow speeds and tail lengths (Fig. 6).

## Discussion

Myliobatid rays generate propulsion primarily through their elongated pectoral fins. Given that myliobatids’ long, slender tails do not produce any thrust during locomotion, it is possible that they serve some alternative hydrodynamic function. In the first study investigating the hydrodynamic role of myliobatid tails, Chaumel et al. (2025) demonstrated that the tail appendage enhances passive stability (i.e., stability achieved without active control or energy input). However, that study only examined models with laterally extended pectoral fins. In reality, myliobatid rays glide with their pectoral fins held in a curved, dihedral position at varying degrees (Supplementary Videos 1–3).

In this study, we evaluate how tail length affects myliobatid gliding stability using models with pectoral fin postures that reflect potential and observed gliding postures (Figs. 1 and 2). Our findings show that, among the dihedral postures (pectoral fins oriented upward relative to the body plane), models with more elevated fin postures (DM3) were less stable than those with lower fin angles (DM1 and DM2), and these effects were most pronounced at high speeds. However, the presence of a tail, regardless of its length, increased stability for all tested fin postures. Tailless models displayed more rolling and pitching movements, greater lateral motion and an elevated ODBA. These results suggest that the long and slender tail of myliobatids increases stability regardless of gliding posture but might be especially important in stabilizing gliding postures with larger dihedral fin angles as seen, for example, in Fig. 1D. This study using models provides the foundation for understanding how tails stabilize the body of myliobatid rays during gliding locomotion.

### Larger pectoral fin angles are less stable

All models were unstable without a tail. However, models with smaller dihedral pectoral fin angles (pectoral fins positioned more horizontally) were more stable than those with larger angles, suggesting that pectoral fin position directly influences stability. Among the DMs, the one with a 6.4° dihedral angle (fin tips slightly tilted upward; DM1) was the most stable, followed by the model with a 35.9° dihedral angle (DM2) and then the 80° dihedral model (DM3). The 80° AM was the least stable of all models tested.

**Fig. 9:**
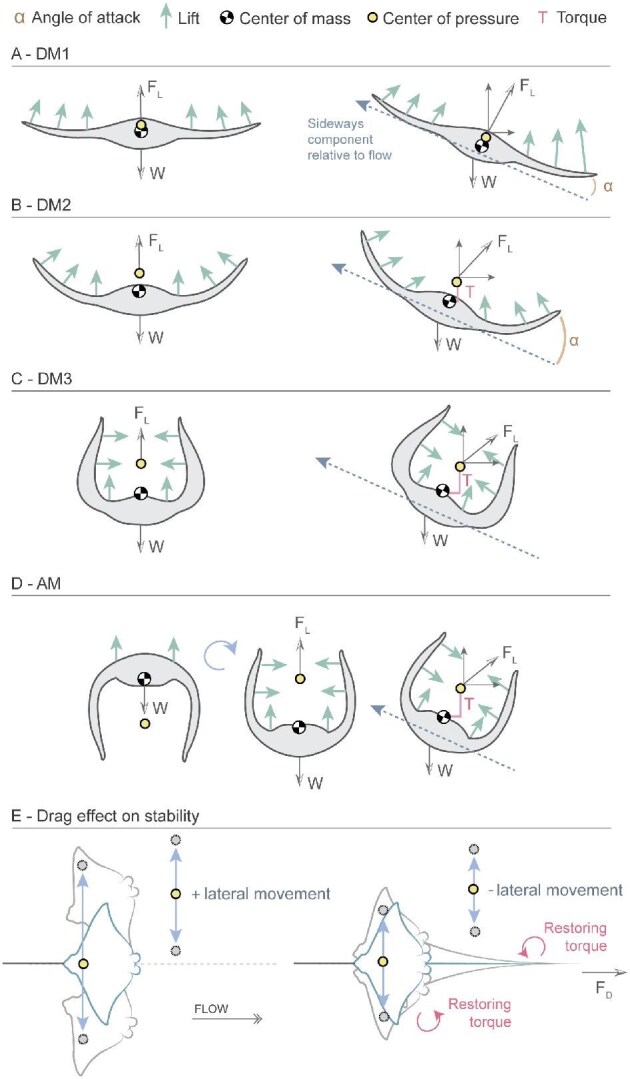
Schematic diagram showing the physical principles occurring in each model tested with the absence of the tail. (A) DM1: Pectoral fins elevated dihedrally at 6.4° create a large projected area, generating strong lift with the COP positioned close to the COM. When the model rolls, the lower fin encounters the flow at a higher angle of attack than the upper fin, producing greater lift and restoring the model to its original position. (B) DM2: Fins elevated at 35.9° reduce the projected area and overall lift while shifting the COP above the COM. During rolling, the lower fin still generates more lift than the upper, restoring the model; however, the smaller active surface reduces vertical lift, and the greater COP–COM separation increases torque, making DM2 less stable than DM1. (C) DM3: Fins elevated dihedrally at 80° provide minimal area exposed to flow, producing little vertical lift. The COP is displaced farther from the COM, increasing torque. When rolling, the fins produce weak vertical lift but stronger horizontal forces, resulting in a less vertically oriented force vector and reduced stability. (D) AM: Fins angled anhedrally at 80° generate negligible lift until higher flow speeds are reached. Once lift occurs, the model flips, placing the COP above the COM. As in DM3, the large COP–COM separation and reduced projected area generate instability. (E) Dorsal view of the model, showing the restoring torque generated by the tail. The tail adds drag and, by generating a restoring torque, it reduces the lateral movement (roll and sway) of the model. Abbreviations: center of pressure (COP), center of mass (COM), drag force (*F*_D_), lift force (*F*_L_), torque (T), weight (W).

In both aerial and aquatic locomotion, the dihedral angle of wings or fins strongly influences lateral stability by (1) altering how lift forces are oriented and distributed between the appendages, and (2) shifting the effective position of the center of mass (COM) (Norberg 1985; Park and Choi 2010; Thomas and Taylor 2001; Harvey and Inman 2021). The ability of a pectoral fin to generate lift depends primarily on two factors: the angle of attack that the fins have toward the flow (greater angles produce more lift) and the projected area (larger areas generate more lift overall) (Fig. 9). When the model is aligned parallel to the flow, the lift force acts vertically. However, when the model rolls laterally, the lift vector tilts with the body, producing both vertical and horizontal components. The vertical lift decreases (less force opposing gravity), while the horizontal lift increases (a component directed sideways), creating a sideways resulting force that makes the model sideslip in the same direction. With straight fins (0°), there is no passive restoring force, so the model slips further toward the rolled side unless actively corrected (by musculature in animals or by pilots in aircraft). By tilting the fins upward in a dihedral angle, the model will become more laterally stable. When the model tilts, as a result of this sideways slip, the model is subjected to a sideways component of the relative flow. During roll-induced sideslip, a dihedral angle presents the lower fin to the flow with a greater angle of attack. It therefore produces more lift than the upper fin, applying a moment in the opposite direction of the roll. And, as a consequence, the model roll back to its original position (Fig. 9A).

Small dihedral angles (DM1) orient the lift vectors so that these asymmetries generate strong restoring moments, passively returning the body toward equilibrium. Moderate dihedral angles (DM2) still produce a restoring moment, but the inward-tilted fins redirect more of the lift vertically rather than laterally. As a result, the ability to oppose roll is weakened. In addition, the steeper fin orientation makes lift vectors more sensitive to small changes in flow or body angle, rendering stability less predictable and requiring more active control during gliding (Fig. 9B). Large dihedral angles (DM3) accentuate this effect: the lift vectors tilt so far inward that the lateral restoring component becomes minimal, or in extreme cases, lift generation is severely compromised due to the reduction of the projected area (Sachs and Moelyadi 2010). Instead of damping roll, these postures can actually amplify it, rendering the system inherently more unstable (Fig. 9C).

The instability of models with high dihedral pectoral angles may also arise from the relative positions of the COM and the center of pressure (COP) (Fig. 9). As the pectoral fins are lifted above the body plane, the COP shifts dorsally relative to the COM. When the COP lies above the COM at a large distance, it generates a larger torque, where small perturbations amplify rather than correct, leading to unstable equilibrium (Fath et al. 2023; Di Santo et al. 2025). The AM model represented the most extreme case. Before lifting off the bottom, the COM was initially below the COP, which would normally favor static stability. However, in this configuration the ventral placement of the pectoral fins prevented them from generating effective lift. As the model responded to flow, it flipped upside-down so that the fins could generate lift, but this inversion also placed the COM above the COP. Thus, the model could only produce lift in a configuration that was inherently unstable, explaining why AM exhibited the greatest instability.

Despite the reduced stability associated with higher pectoral fin angles, gliding myliobatids, as well as birds can position their pectoral fins (or wings) at different angles depending on their locomotor needs (Supplementary Videos 1–3). While stability is classically thought to be desirable, however, instability can be beneficial by enhancing maneuverability (Thomas and Taylor 2001; Sachs and Moelyadi 2010; Durston 2019; Fath et al. 2023; Di Santo et al. 2025). For example, birds that rely on agile flight, such as owls, peregrine falcons, and swifts, often glide with anhedral or large dihedral wing positions (Thomas and Taylor 2001; Lentink et al. 2007; Sachs and Moelyadi 2010; Durston 2019; Usherwood et al. 2020). Similarly, myliobatids occasionally use pronounced dihedral gliding positions (similar to DM3). For example, cownose rays (*Rhinoptera bonasus*) exhibit extreme/dramatic dihedral in glides when facing an oncoming wave (Fish and Hoffman 2015), which may indicate that large dihedral angles are better when facing turbulent flows. Spotted eagle rays (*Aetobatus narinari*) have also been observed gliding with this fin posture (Supplementary Video 2). During glides with extreme dihedral fin positions, myliobatids such as *A. narinari* or *R. bonasus* can take advantage of being more unstable as small corrections in the position of the pectoral fins or the fin tips can produce considerable variation in body position (i.e., be more maneuverable), which may be also advantageous in conserving energy, as small corrections can correct heading. Additionally, these species may use other control surfaces, such as pelvic fins, to improve stability and modulate body orientation.

In addition to fin posture, speed was an important component that affected stability. Gliding behavior in myliobatids has been associated with swimming speeds below 1 Bl s^−1^ (Braun et al. 2014; Fish et al. 2016, 2018). The fastest myliobatids are known to swim at speeds of above 4 m s^−1^ (2.2 Bl s^−1^) based on the average Bl of the giant manta ray, *Mobula birostris*, according to Fish et al. (2016), though swimming speeds under 1 Bl s^−1^ are common for long distance swimming, foraging, and gliding (Parson et al. 2011; Fontanella et al. 2013; Fish et al. 2016). Additionally, mantas use passive gliding to complete turns at swimming speeds between 0.4 and 1.1 Bl s^−^^1^ (Parson et al. 2011). The flow speeds evaluated in this study (1.1–6.0 Bl s^−^^1^) included unrealistic swimming speeds, which allowed us to observe model behavior under extreme conditions. Unfortunately, since our models did not generate sufficient lift to overcome bodyweight at speeds below 1.1 Bl s^−1^, our tested flow speeds do not span the full range of gliding speeds observed in living myliobatids. However, the slowest flow speeds tested in this study do represent realistic gliding speeds and our results indicate that tails are key to the stability of models with lower dihedral fin angles (DM1 and DM2) at these speeds. The efficacy of tails in mitigating roll and, by consequence, ODBA for DMs at low flow speeds reinforces the concept that ray tails enable intermittent gliding and support vital slow swimming behaviors like turning and foraging.

### The tail improves stability in gliding model postures

Myliobatids are known to intersperse passive glides within periods of active oscillatory locomotion, where the gliding phase typically follows an upstroke of the enlarged pectoral fins (Parson et al. 2011; Braun et al. 2014; Fish et al. 2018; Fontes et al. 2022). Although gliding may fulfill several key functions (Socha et al. 2015), it is likely performed by myliobatids to reduce energy expenditure during active locomotion, as is the case in numerous other aquatic and terrestrial animals (Baudinette and Schmidt-Nielsen 1974; Williams et al. 2000; Williams 2001; Gleiss, Jorgensen, et al. 2011; Sakamoto et al. 2013; Mizrahy-Rewald et al. 2022). For instance, myliobatid rays are capable of long-distance dives, descending deep into the water column (Braun et al. 2014; Fontes et al. 2022). Since gliding involves passive sinking, it is possible that these rays use gliding as a means to descend efficiently, conserving energy during vertical movements. Maintaining a stable body posture is also critical for energy efficiency while gliding, as inherently unstable animals expend additional energy to counteract rotational torques that disturb their upright orientation (Fath et al. 2023; Di Santo et al. 2025). Thus, animals that glide tend to feature stabilizing and controlling surfaces such as wings positioned in a dihedral angle, tails, feathers, and/or membranes to achieve inherent (or passive) stability.

Our results show that all models were more stable with a tail, suggesting that the tail plays a crucial role in stabilizing models by reducing pitching, rolling, and swaying movements regardless of pectoral fin position. Tails were particularly critical for models with the most unstable pectoral fin postures, such as extreme dihedral or anhedral angles (as in DM3 and AM). Nevertheless, since our tests were conducted with rigid, tethered models, several experimental limitations must be considered. Real myliobatid rays are flexible, free-swimming animals, that can alter their body confirmation in response to stimuli, and whose morphology differs across species (Rosenberger 2001; Last 2016; Chaumel et al. 2025). Our tethered set-up constrained pitching rotation, which is important for gliding. However, several studies have demonstrated the value of using tethered models to test both aerodynamic and hydrodynamic effects of control or stabilizing surfaces as this testing arrangement allows for detailed measurements of roll, sway, and ODBA as well as high-speed video.

The stabilizing effect of the tail in our models is consistent with the *kite hypothesis* proposed by Chaumel et al. (2025), which tested models with extended pectoral fins. According to the kite hypothesis, myliobatid tails act like a tail of a kite, increasing drag posterior to the COM and pressure, which induces a restoring torque that limits pitching, rolling, and yawing movements (Marvin 1897). Tails longer than 0.9 Bl effectively dampen lateral movements, whereas shorter tails are unable to restore the model to its original position following a perturbation. As such, myliobatids tails act as a control surface (Fish and Lauder 2006, 2017), stabilizing the free body by adding drag posterior to the COM, instead of generating lift. Our findings corroborate this notion and demonstrate that the tail is especially important in body postures with larger dihedral fin angles, which are inherently less stable. Chaumel et al. (2025) reported that myliobatids typically possess tails ranging from 0.9 to 4.6 Bl, further evidence that the tail plays a functional role as a stabilizing surface in real animals. Consistent with their findings, we also observed that longer tails (∼3× the Bl) did not provide additional stabilizing benefits for any of the pectoral fin configurations. This implies that the tail likely serves other functions in species with exceptionally long tails, such as providing mechanosensory input, increasing hydrodynamic efficiency, or participating in conspecific communication as, for example, during mating (Chaumel and Lauder 2025).

The stabilizing effect of tails during gliding has been widely described in other animals and structures, including birds, fishes, kites, and airplanes (Hummel 1992; Thomas and Taylor 2001; Sachs 2007; Durston 2019; Fish et al. 2021). However, unlike birds, which can actively manipulate tail morphology to meet different aerodynamic demands (e.g., spreading the tail feathers to generate lift, trimming it to reduce drag, or angling it upward for stability) (Hummel 1992; Thomas and Taylor 2001; Aldheeb et al. 2016), the whip-tail of myliobatids has limited mobility (Chaumel and Lauder 2025). Although myliobatids possess musculature and segmented vertebrae at the base of the tail, the main part of the tail has a reduced or lacks musculature and is supported by fused vertebrae along its length (Chaumel and Lauder 2025). Consequently, despite some active movement at the tail base, the whip-like tail primarily exhibits passive motion trailing behind the body. We propose that the whip-like tail instead functions as a passive stabilizing control surface during gliding, enhancing locomotor efficiency by reducing the energetic cost of active stabilization and thereby increasing locomotor efficiency. In the absence of a tail, myliobatids would be less stable and would likely need to expend more energy to maintain control during gliding. Similarly, myliobatid inspired autonomous underwater vehicles may benefit from the addition of biomimetic tails.

## Supplementary Material

obag002_Supplemental_Files

## Data Availability

Biologger raw data are publicly available at Mendeley Repository. https://data.mendeley.com/datasets/95syyp422h/1
